# In-House 3D-Printed Surgical Guides with a Minimally Invasive Design for Asymmetric Mentoplasty: A Case Series

**DOI:** 10.3390/dj14030135

**Published:** 2026-03-02

**Authors:** Guilherme Pivatto Louzada, Bianca Pulino, Raissa Dias Fares, Ana Beatriz Goettnauer de Cerqueira, Diogo de Vasconcelos Macedo, Guilherme Zanovelli Silva, Thiago Nunes Palhares, Gustavo Câmara, Heloisa Marão, Marcella Bonfim, Henrique Furukawa, Jamil Shibli, Raphael Capelli Guerra

**Affiliations:** 1 Hospital Sírio-Libanês, Instituto de Ensino e Pesquisa, São Paulo 01308-050, Brazilgustavoqcamara@gmail.com (G.C.);; 2 Hospital Municipalizado Adão Pereira Nunes, Rio de Janeiro 25213-020, Brazil; 3 Marinha do Brasil, Odontocliinica Central do Rio de Janiero, Rio de Janeiro 20091-000, Brazil; 4 Department of Oral and Maxillofacial Surgery, Faculdade Israelita de Ciências da Saúde, São Paulo 05652-900, Brazil; 5 Department of Oral and Maxillofacial Surgery, Hospital Israelita Albert Einstein, São Paulo 05652-900, Brazil; 6 Department of Oral and Maxillofacial Surgery, Universidade Estadual Paulista, Araraquara 14801-385, Brazil; 7 Centro de Tecnologia de Informação o Renato Archer, Campinas 13069-901, Brazil; 8 Department of Periodontology, Universidade Santo Amaro, São Paulo 04829-300, Brazil; 9 Department of Peridontology, Universidade Guarulhos UNG, Guarulhos 07023-070, Brazil

**Keywords:** asymmetric mentoplasty, genioplasty, 3D printing, surgical guides, minimally invasive design, case series

## Abstract

**Background**: Mentoplasty plays a central role in facial harmony, particularly in the correction of chin asymmetries. Advances in digital planning and three-dimensional (3D) printing have enabled the development of patient-specific surgical guides; however, their clinical implementation in public healthcare settings remains limited by workflow complexity and accessibility. **Methods**: This case series describes three patients who underwent asymmetric mentoplasty using customized, in-house-manufactured 3D-printed cutting and positioning guides. Virtual surgical planning was performed using cone-beam computed tomography (CBCT) data, followed by guide design and fabrication through an in-house digital workflow. Postoperative CBCT was used for descriptive assessment of the correspondence between planned and achieved chin positions using predefined linear reference points. **Results**: Across the three cases, postoperative CBCT analysis demonstrated encouraging agreement between virtual planning and achieved surgical outcomes. The customized guides facilitated accurate transfer of the planned osteotomy and chin positioning in both symmetric and asymmetric movements. No major intraoperative or postoperative complications were observed. Guide fabrication required low material consumption, enabling rapid in-house production with minimal direct material cost. **Conclusions**: Within the limitations of a small case series, the proposed in-house digital workflow demonstrated technical feasibility and encouraging agreement between planned and achieved chin positioning in selected asymmetric mentoplasty cases. Further studies with larger samples and standardized accuracy assessment methods are required to confirm these preliminary observations.

## 1. Introduction

The chin, or mentum, is a significant component of facial aesthetic balance [1]. In this manuscript, the term “mentoplasty” is used interchangeably with “genioplasty,” in accordance with current maxillofacial surgery literature. It represents the projecting portion of the mandibular symphysis and constitutes a distinctive anatomical feature in humans [2]. Mentoplasty is a surgical procedure aimed at improving chin morphology and facial harmony by addressing soft tissue deficiency, the mento-cervical angle, horizontal or vertical discrepancies, and, most notably, facial asymmetry, which remains one of the main challenges in chin correction procedures [3,4].

Mentoplasty can be broadly classified into two main categories: augmentation and reduction. Augmentation procedures increase chin projection using implants or bone repositioning, whereas reduction procedures aim to decrease chin prominence by osteotomy and repositioning techniques [4,5]. Over the past decade, three-dimensional (3D) printing has emerged as an important tool in maxillofacial surgery, enabling patient-specific planning and the fabrication of customized surgical devices. The development of computer-aided surgical simulation (CASS) has allowed surgeons to virtually plan osteotomies and design surgical guides tailored to individual anatomy [5].

Surgical guides can be classified as pre-formed or in-house manufactured. The latter, which represent the focus of the present study, include tooth-supported or bone-supported guides and customized fixation plates designed to assist osteotomy execution and segment repositioning. Patient-specific guides improve control over asymmetric osteotomies and facilitate accurate transfer of virtual planning to the operative field. In addition, compact and strategically supported guide designs may facilitate placement under limited surgical access, reducing the need for extensive exposure while preserving positional fidelity [6]. Tooth-supported guide designs incorporating predefined screw positions provide stable and reproducible positioning while minimizing the risk of intraoperative displacement. However, conventional drilling and cutting guides often require wide surgical exposure to allow proper seating and fixation, which may increase soft tissue detachment [7].

Although minimally invasive concepts have been discussed in the context of orthognathic surgery, the present work does not aim to demonstrate or validate a minimally invasive surgical technique. Instead, the design concept applied to the surgical guides focused on optimizing guide geometry and support to facilitate placement under limited surgical access, independent of the specific mentoplasty technique employed, including conventional or mini-wing osteotomies [7,8,9,10,11].

Therefore, the aim of this study was to report a case series describing the feasibility of an in-house digital workflow for the design and fabrication of customized 3D-printed cutting and positioning guides for the correction of chin asymmetry. This exploratory report focuses on workflow description, technical feasibility, and clinical applicability, without the intention of providing generalizable conclusions or validating surgical accuracy beyond descriptive observations.

## 2. Cases Series

This manuscript is reported as a descriptive case series. The authors declare that the work complied with the Code of Ethics of the World Medical Association (Declaration of Helsinki) and the Recommendations for the Conduct, Reporting, Editing, and Publication of Scholarly Work in Medical Journals. The patients were aged between 20 and 28 years, were systemically healthy, and presented no contraindications for elective surgical procedures. This case report study was approved by the Institutional Ethics Committee from Centro Universitário Serra dos Órgãos (UNIFESO), Teresópolis-Rio de Janeiro/Brazil, under the protocol number (CAAE: 88434125.9.0000.5247). This case series is reported in accordance with the CARE (CAse REport) guidelines.

### 2.1. Study Design and Stepwise Workflow (Protocol)

**Figure 1:**
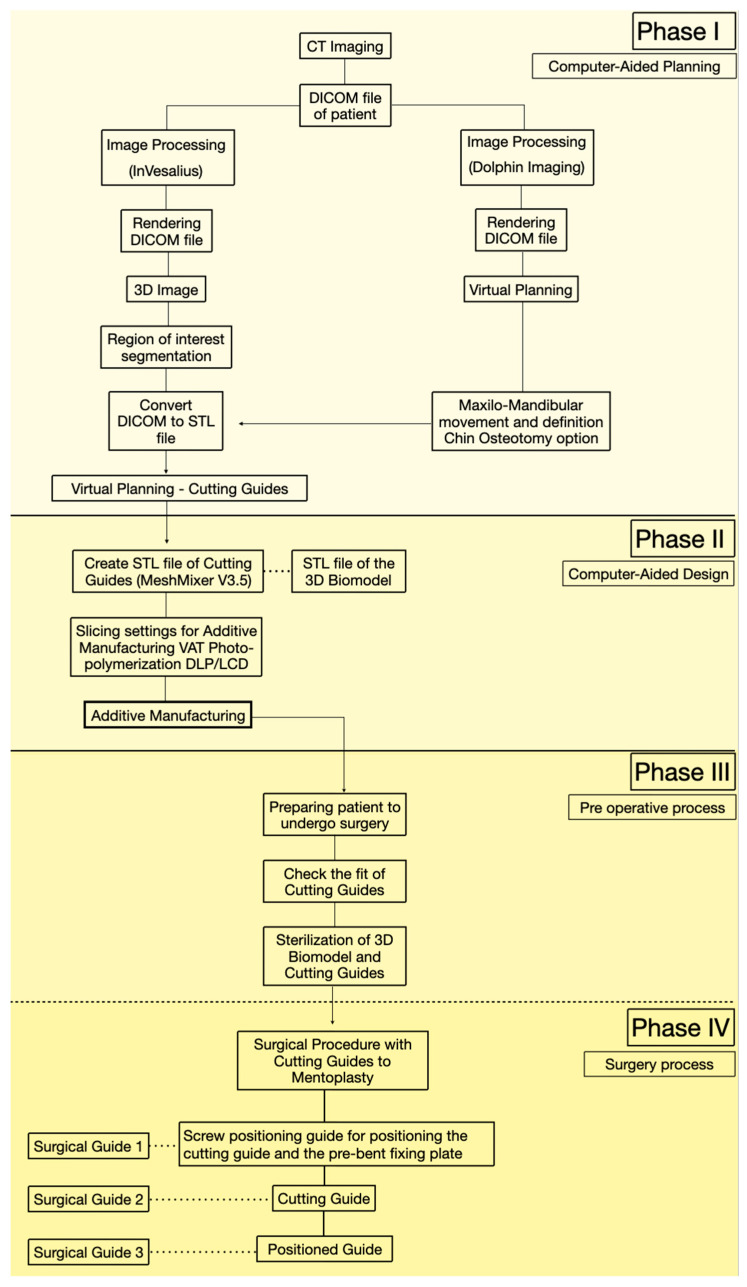
Workflow of virtual planning, guide fabrication, and three-stage surgical sequence.

This manuscript is presented as a case series describing an in-house digital workflow for designing and manufacturing patient-specific cutting and positioning guides with a minimally invasive design for asymmetric mentoplasty. All cases were managed within the same institutional setting and followed a standardized protocol. The purpose of this section is to provide a reproducible, stepwise sequence of data acquisition, virtual planning, guide design/printing, verification, sterilization, and intraoperative use. The overall workflow is summarized in Figure 1.

**Step 1—Imaging acquisition and digital records (CBCT/DICOM and dental scans)**

**Figure 2:**
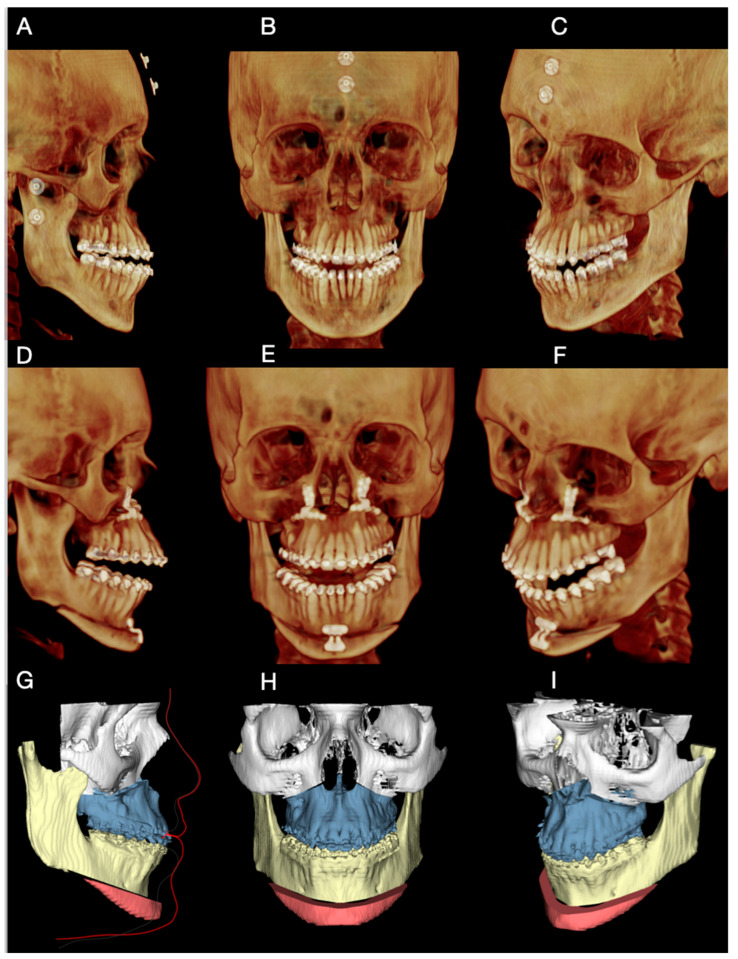
Preoperative imaging, virtual planning, and postoperative assessment in Case 1. (**A**–**C**) Preoperative CBCT images acquired in natural head position. External metallic skin markers were temporarily placed to assist in visual verification of head orientation during virtual planning. (**D**–**F**) Postoperative CBCT images obtained at 6 months, demonstrating the achieved chin position. (**G**–**I**) Virtual surgical planning images illustrating the final planned position of the chin segment and the mini-wing osteotomy design.

Each patient underwent cone-beam computed tomography (CBCT) acquisition in natural head position, generating DICOM data (Digital Imaging and Communications in Medicine). Preoperative CBCT examples are shown in Figure 2A–C (Case 1). In parallel, intraoral digital scans of the maxillary and mandibular arches were obtained separately and then registered in the final occlusal position, generating three STL files (maxilla, mandible, and occlusion record).

**Step 2—Head position standardization (double-check strategy)**

To improve consistency between acquisition and planning, head orientation was controlled using a double-check approach:

1. External skin markers: when CBCT acquisition was performed immediately after marker placement, external skin markers were used as an auxiliary visual reference to confirm head orientation within the planning environment (Figure 2A–C) Their role was to support verification rather than to be mandatory for all cases.
2. Digital photographic alignment: when markers were not available or CBCT was performed at an external imaging center, head orientation was standardized digitally by aligning the CBCT dataset according to the patient’s extraoral photograph. This was performed using a screen-capture from the virtual planning environment and image overlay with the extraoral photograph to visually match facial reference planes. This second step functioned as a reproducibility safeguard to maintain the planned head position.

**Step 3—Virtual surgical planning (VSP)**

DICOM and STL datasets were imported and integrated in Dolphin Imaging software, version 11.9^®^ (Dolphin Imaging & Management Solutions, Chatsworth, CA, USA) to create a composite 3D skull model. Virtual planning included: (i) osteotomy design and position; (ii) definition of the intended chin movement (linear and/or asymmetric components); and (iii) generation of a final STL model representing the planned postoperative chin position (Figure 2G–I).

**Step 4—Guide design (access-optimized design) and software environment**

The final skull/mandible STL file was exported and transferred to Meshmixer, version 3.5 (Autodesk Inc., San Rafael, CA, USA) for guide design. Initially, the mandibular surface corresponding to the intended guide contact area was isolated using the selection and sculpting tools to avoid undercuts and areas that would hinder guide seating under limited surgical exposure. A surface offset was applied to generate an intimate but non-interfering bone contact, preserving passive seating.

Guide thickness was standardized to ensure mechanical stability while maintaining a compact profile compatible with reduced surgical access. Screw access paths were planned perpendicular to the bone surface at predefined locations to allow secure fixation without encroaching on the osteotomy line.

Guides were intentionally designed to be compact, segmented when required, and strategically supported, aiming to facilitate placement under limited surgical exposure (access-optimized design), independent of the specific mentoplasty technique employed (conventional, mini-wing, etc.).

**Figure 3:**
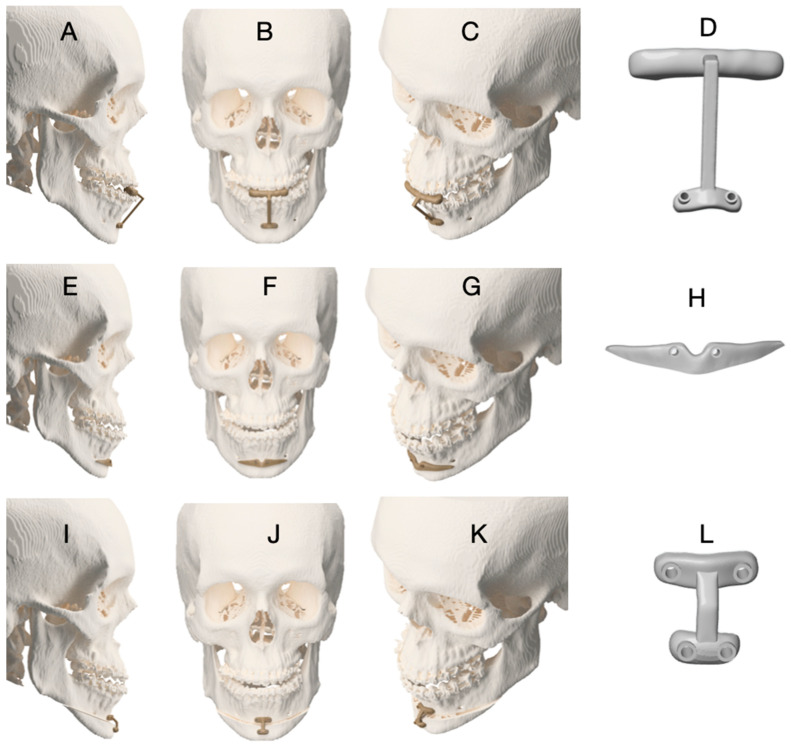
Sequence of customized surgical guides used for asymmetric mentoplasty in Case 1. (**A**–**D**) Guide 1: Occlusal-supported drilling orientation guide, used to define and standardize the position of the pilot holes that serve as reference for the subsequent cutting guide. (**E**–**H**) Guide 2: Bone-supported cutting guide, used to mark the osteotomy lines on the mandibular symphysis according to the virtual surgical plan. (**I**–**L**) Guide 3: Chin positioning guide designed to transfer the planned final position of the menton segment. To ensure accurate positioning, the guide is placed and pilot holes are pre-drilled before complete separation of the chin segment, preserving the predefined screw trajectory after mobilization and allowing faithful reproduction of the virtual plan.

A three-guide sequence was adopted:

- **Guide 1 (occlusal-supported drilling orientation guide):** provides a stable dental reference to reduce cumulative error when surgical exposure is limited. The occlusal reference helps ensure reproducible alignment before bone-supported guide seating.
- **Guide 2 (bone-supported cutting/marking guide):** used to define osteotomy orientation and mark the cutting line (Figure 3A–H).
- **Guide 3 (positioning guide):** designed to transfer the planned final chin segment position (Figure 3I–L). To increase fidelity, the positioning guide workflow included pilot-hole marking/pre-drilling before complete segment separation, preserving the screw trajectory after mobilization (as also detailed in Figure 3 legend). The functional role of each guide in the three-step sequence is illustrated in Figure 3. Briefly, the occlusal-supported guide (Guide 1) establishes a stable dental reference for standardized pilot hole orientation; the bone-supported cutting guide (Guide 2) transfers the planned osteotomy lines to the mandibular symphysis; and the positioning guide (Guide 3) is used to transfer the planned final position of the chin segment by preserving the predefined screw trajectory prior to complete segment mobilization. This sequential logic aims to reduce cumulative positioning error while maintaining compatibility with limited surgical access.

Boolean operations were used to subtract the mandibular surface from the guide body, and cylindrical drill paths were generated to guide screw placement. Each guide was visually inspected within the software to confirm seating, screw trajectory alignment, and absence of interference before exporting the final STL files for fabrication.

Contact surfaces were intentionally limited to stable cortical regions and designed to avoid engagement with anatomical undercuts, reducing the risk of forced seating or deformation during intraoperative placement. Acceptance of each guide design was based on the absence of visible gaps at the contact interface, passive alignment of screw trajectories, and the ability to position the guide digitally without deformation or interference.

**Step 5—Additive manufacturing (printing) and preoperative model**

Patient-specific guides were printed using vat photopolymerization (DLP/LCD) on a desktop printer (Anycubic^®^; Shenzhen Anycubic Technology Co., Ltd., Shenzhen, China). Slicing and print preparation were performed using Anycubic Photon Workshop software, version 3.6.2 (Shenzhen Anycubic Technology Co., Ltd., Shenzhen, China). A patient-specific mandibular model (with final planned chin position) was also printed for preoperative verification and plate adaptation.

**Step 6—Material identification, post-processing, and debris mitigation measures**

All guides were fabricated using a biocompatible photopolymer resin indicated for surgical guide manufacturing (priZma 3D Bio Guide, MakertechLabs, Rio de Janeiro, Brazil; ANVISA No. 80483740003), designed for DLP/LCD systems and indicated for implant guides, periodontal surgical guides, orthognathic splints, and related applications.

Post-processing followed a standardized protocol: 99% isopropyl alcohol wash (5 min) to remove residual resin, followed by UV post-curing (405 nm, 10 min) to finalize polymerization and improve mechanical properties. In addition, to reduce the risk of polymer debris during cutting, the cutting guide was designed with a short profile that guides the initial trajectory of osteotomy rather than the full depth of the cut, minimizing unintended blade-to-guide contact. Purple resin coloration was used to facilitate intraoperative visual identification of any potential debris, if present.

**Step 7—Sterilization and post-sterilization verification (fit and stability)**

All guides were sterilized using low-temperature hydrogen peroxide plasma sterilization (Sterrad system; ~45 min cycle, 50 °C). Sterility indicators were used to confirm cycle completion. After sterilization, each guide was re-tested on the corresponding patient-specific 3D-printed mandibular model to verify fit and detect potential distortion before intraoperative use.

Prior to surgery, all guides underwent a verification step on the corresponding patient-specific 3D-printed mandibular model. Guide fit was considered acceptable when the following criteria were met:

(i) Stable seating without rocking or visible gaps at the intended contact points;
(ii) Passive adaptation without the need for forceful positioning.
(iii) Correct alignment of predrilled screw trajectories with the planned osteotomy and positioning paths. Any guide that did not meet these acceptance criteria was redesigned and reprinted before clinical use.

**Step 8—Intraoperative sequence and acceptance criteria (guide seating and fixation)**

Guide seating was accepted when:

(i) **Guide 1** showed stable seating without rocking;
(ii) bone-supported guides showed intimate adaptation at planned contact points without soft tissue interposition; and
(iii) screw access paths were passively aligned without forcing.

**Guide 1** was used to orient initial drilling, followed by fixation and use of **Guide 2** for osteotomy marking (Figure 3A–H). Osteotomy marking could be performed using Piezosurgery^®^ OT12 Micro Saw (Mectron S.p.A., Carasco, Italy) or other cutting instruments depending on surgeon preference. After marking, the osteotomy was completed as planned. For **Guide 3**, pilot holes were marked/pre-drilled before complete segment separation to preserve screw trajectory and improve fidelity of final positioning (Figure 3I–L). Final fixation method (plate and/or screws) was selected according to osteotomy design and case-specific requirements.

**Step 9—Postoperative assessment and follow-up**

Postoperative CBCT at 6 months was used to assess correspondence between planned and achieved chin position (Figure 2D–F). Standardized clinical photographs were obtained at 12 months to document frontal and profile outcomes. Operative time and perioperative events (including sensory changes) were recorded for each case and reported in the Results.

### 2.2. First Case

Case 1 involved a 20-year-old male patient, systemically healthy, with no relevant medical comorbidities, who sought correction of chin asymmetry and a reduced cervicomental angle. Frontal examination revealed mild mandibular base asymmetry, without deviation of the mandibular plane or dental midline.

Preoperative CBCT imaging and extraoral clinical photographs were used for diagnostic assessment and virtual planning (Figure 2A–C). Given the favorable sagittal mandibular position, the surgical plan focused exclusively on chin advancement. A mini-wing advancement mentoplasty was planned to increase mandibular body projection, enhance transverse facial balance, and achieve a more squared chin contour, considered aesthetically desirable for male patients. A slight rightward displacement of the chin segment was also planned to correct the preexisting asymmetry, as defined during virtual surgical planning (Figure 2G–I).

The surgical procedure followed the standardized digital workflow described in Section 2.1. An occlusal-supported drilling guide **(Guide 1)** was first positioned to establish a stable dental reference, followed by a bone-supported cutting guide **(Guide 2)** to mark the osteotomy lines (Figure 3A–D). The osteotomy was subsequently completed using piezosurgical and reciprocating instruments after guide removal (Figure 3E–H). Although the mini-wing osteotomy is more extensive than conventional mentoplasty techniques, minimally invasive principles were applied, including tunneling access and limited soft tissue detachment. These measures aimed to reduce periosteal stripping while preserving surgical accuracy.

**Figure 4:**
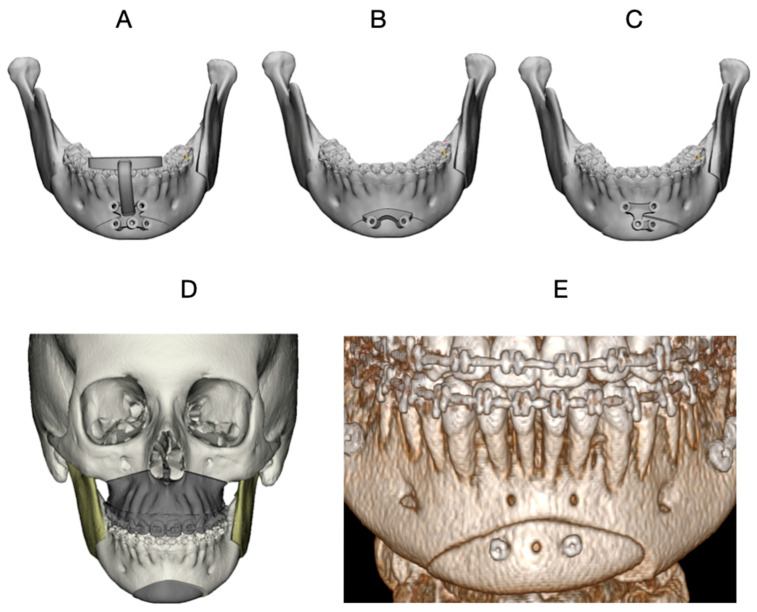
Customized surgical guides and virtual planning for asymmetric mentoplasty in Case 2. (**A**) Guide 1: Occlusal-supported drilling orientation guide, used to standardize the position of pilot holes for subsequent guide placement. (**B**) Guide 2: Bone-supported cutting guide used to mark the planned osteotomy lines on the mandibular symphysis. (**C**) Guide 3: Chin positioning guide used to transfer the planned final position of the menton segment according to the virtual surgical plan. (**D**) Virtual surgical planning illustrating maxillomandibular movements and the asymmetric chin repositioning. (**E**) Postoperative CBCT obtained at 6 months, demonstrating the achieved chin position.

Final fixation was achieved using a pre-bent advancement plate. Although lag screw fixation was not employed in this case, a positioning guide (Guide 3) was intentionally used as a transfer guide to reproduce the virtually planned chin position before definitive plate fixation (Figure 4A–C). Guide 3 was positioned prior to complete mobilization of the menton segment to allow accurate marking and preservation of the planned spatial relationship. This step improved the fidelity of final positioning and reduced the risk of intraoperative deviation during plate adaptation, even when definitive fixation relied exclusively on plate osteosynthesis (Figure 4A–C).

**Figure 5:**
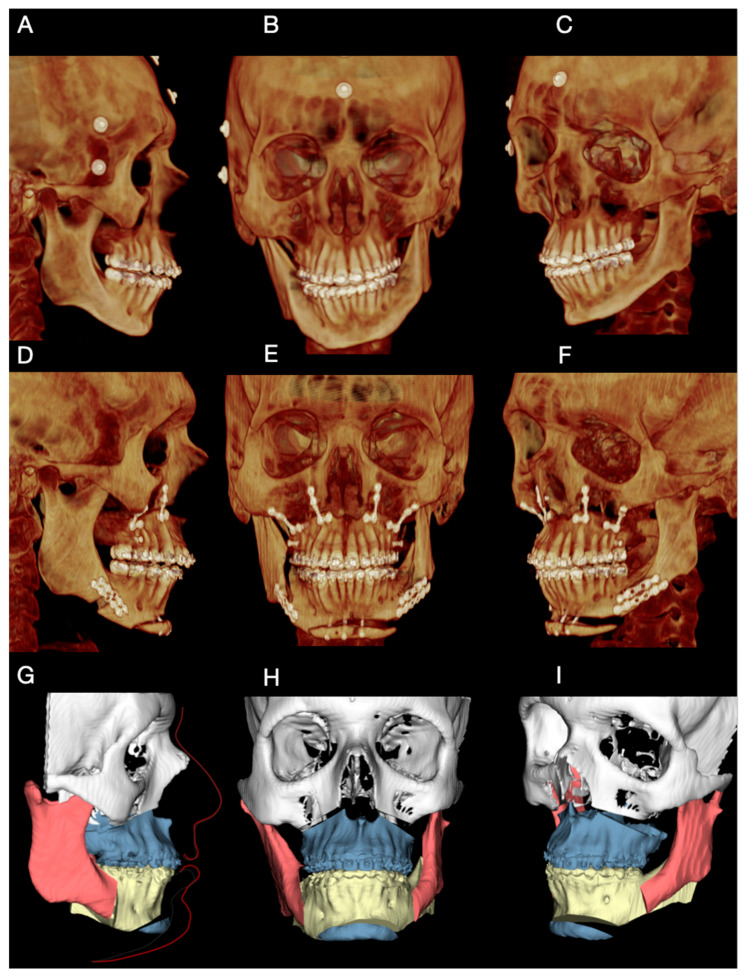
Preoperative imaging, virtual planning, and postoperative evaluation for Case 3. (**A**–**C**) Preoperative CBCT images acquired in natural head position. External metal skin markers (metal spheres) were temporarily fixed to the skin surface to assist in reproducing head orientation during virtual planning. (**D**–**F**) Postoperative CBCT images obtained at 6 months, demonstrating the achieved mandibular and chin position. (**G**–**I**) Virtual surgical planning images illustrating the planned maxillomandibular movements and the asymmetric repositioning of the chin segment, highlighting the relationship between the planned correction and the final chin position.

The total operative time for the mentoplasty procedure was approximately 45 min, measured from mucosal incision to final wound closure. Postoperative CBCT obtained at 6 months demonstrated close correspondence between the planned and achieved chin position (Figure 5A–C). At the 12-month follow-up, clinical photographs showed correction of frontal asymmetry and improved chin projection and profile balance in the lateral view.

### 2.3. Second Case

Case 2 involved a 22-year-old female patient, systemically healthy, with no relevant medical comorbidities, who sought correction of chin asymmetry. Based on the limited magnitude of the planned asymmetric movement, a compact surgical guide design was selected to facilitate placement under reduced surgical access when compared with larger positioning guides.

The surgical sequence followed the standardized workflow described in Section 2.1. No large translational movements were required, and fixation was achieved using two screws after completion of the planned osteotomy. The use of patient-specific guides aimed to improve predictability in asymmetric corrections, despite requiring careful adaptation during placement. The operative time for this procedure was approximately 40 min. The reduced surgical exposure and limited magnitude of movement contributed to a shorter operative duration compared to Case 1.

Postoperative clinical photographs demonstrated appropriate chin positioning in the frontal view) and balanced lower facial thirds in the profile view). The osteotomy design followed standard genioplasty safety principles, maintaining appropriate anatomical margins relative to the inferior alveolar nerve. Postoperatively, only mild and transient sensory alterations were observed, with rapid recovery during the first postoperative month. Although the postoperative CBCT demonstrated a slight difference in the orientation of the chin segment when compared with the virtual surgical plan, this minor rotational variation did not compromise the intended clinical outcome. Such discrepancies are expected in asymmetric mentoplasty cases with limited magnitude of movement and reflect soft tissue adaptation and fixation dynamics rather than inaccurate guide transfer. Importantly, the final chin position achieved the planned correction of asymmetry and satisfactory facial balance.

### 2.4. Third Case

Case 3 involved a 28-year-old male patient, systemically healthy, with no relevant medical comorbidities, who sought correction of severe laterognathism associated with left temporomandibular joint (TMJ) hypoplasia, within a broader spectrum of skeletal facial asymmetry (Figure 5A–C). Imaging demonstrated temporomandibular joint hypoplasia without active degenerative changes, and the patient was clinically asymptomatic with preserved joint function.

Given the complexity of the facial asymmetry and the presence of a retrognathic profile, a combined orthognathic approach was planned, including bimaxillary surgery associated with asymmetric mentoplasty (Figure 5G–I). Asymmetric chin repositioning represents a surgical challenge due to the lack of stable intraoperative references for precise fixation, reinforcing the role of cutting and positioning guides in transferring the virtual plan to the operative field.

**Figure 6:**
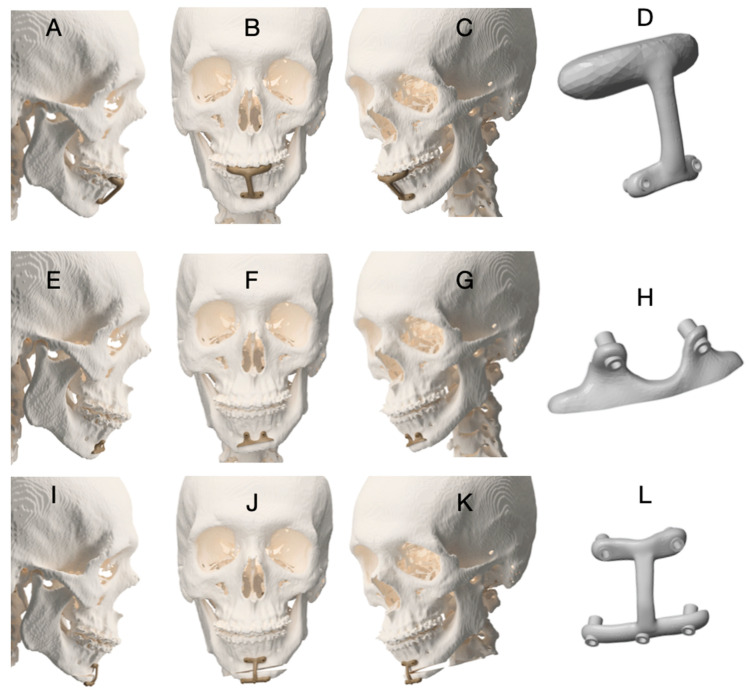
Sequential use of surgical guides for asymmetric mentoplasty in Case 3. (**A**–**D**) Guide 1 (occlusal-supported drilling guide) used to orient pilot-hole drilling and ensure accurate positioning of the subsequent bone-supported guide. (**E**–**H**) Guide 2 (bone-supported cutting guide) used to mark and guide the osteotomy lines of the chin segment. (**I**–**L**) Guide 3 (positioning guide) used to transfer the planned chin position according to the virtual surgical plan. Pilot holes were pre-drilled in the inferior portion of the chin segment before complete mobilization to preserve the predefined screw trajectory, thereby improving the fidelity of final fixation and positioning.

The same guide sequencing protocol described in Case 1 was applied. The surgical sequence (Figure 6A–L) shows that mentoplasty was intentionally performed before maxillary advancement and bilateral sagittal split osteotomies. This strategy was adopted to avoid biomechanical overload on the chin segment during subsequent skeletal repositioning, thereby minimizing the risk of displacement or loss of accuracy in the planned chin position.

In this case, the positioning guide was designed with reduced dimensions to correspond to a smaller osteotomy compared with Case 1. After placement of Guide 3, temporary fixation screws were used to stabilize the chin segment, followed by definitive fixation according to the virtual surgical plan. Postoperative CBCT performed at 6 months demonstrated close agreement between the planned and achieved chin position.

Clinical photographs revealed a marked improvement in frontal facial symmetry. In cases of severe skeletal asymmetry, soft tissue adaptation may be incomplete, and optimal bone repositioning does not necessarily result in total facial symmetry. Despite mild residual frontal asymmetry, patient-reported satisfaction was high, as assessed using a visual analog scale (VAS).

**Figure 7:**
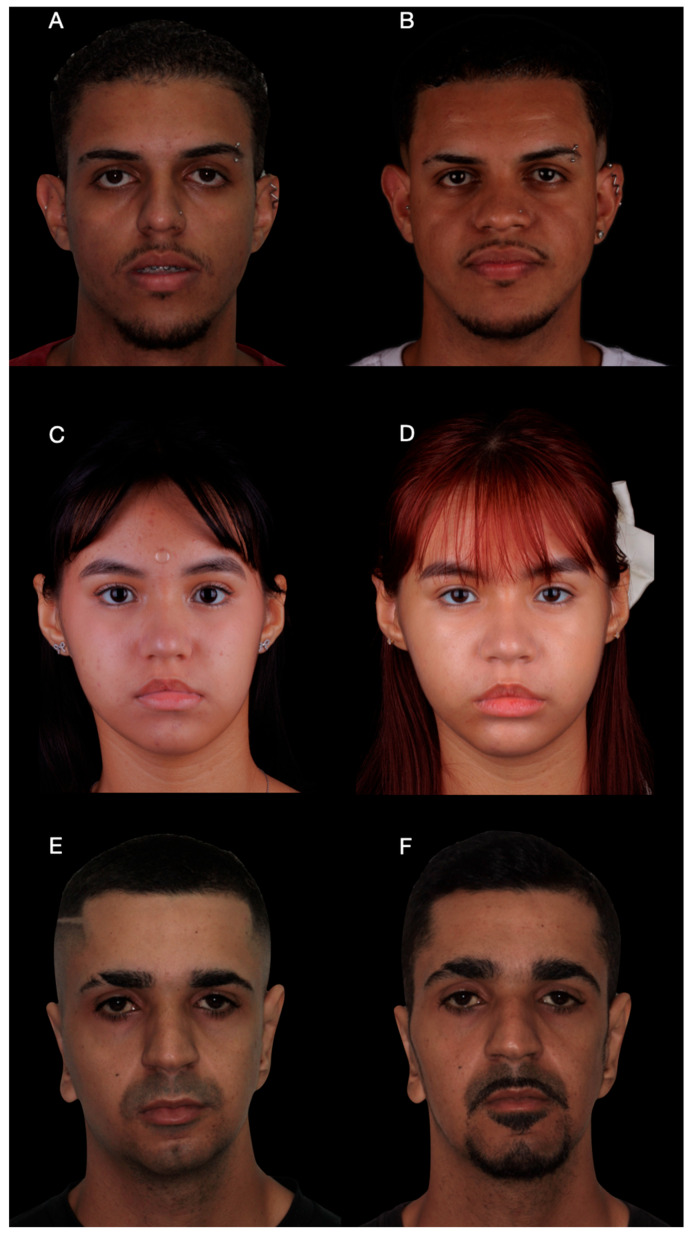
Frontal clinical photographs before and after surgery. (**A**,**B**) Patient 1, frontal view before surgery and at 12-month follow-up, demonstrating correction of chin asymmetry. (**C**,**D**) Patient 2, frontal view before surgery and at 12-month follow-up, showing improved lower facial symmetry. (**E**,**F**) Patient 3, frontal view before surgery and at 12-month follow-up. Mild residual asymmetry of the lower face is observed, particularly related to soft tissue characteristics. Despite this, an evident improvement in chin positioning and overall facial balance was achieved, corresponding to the primary surgical objective.

**Figure 8:**
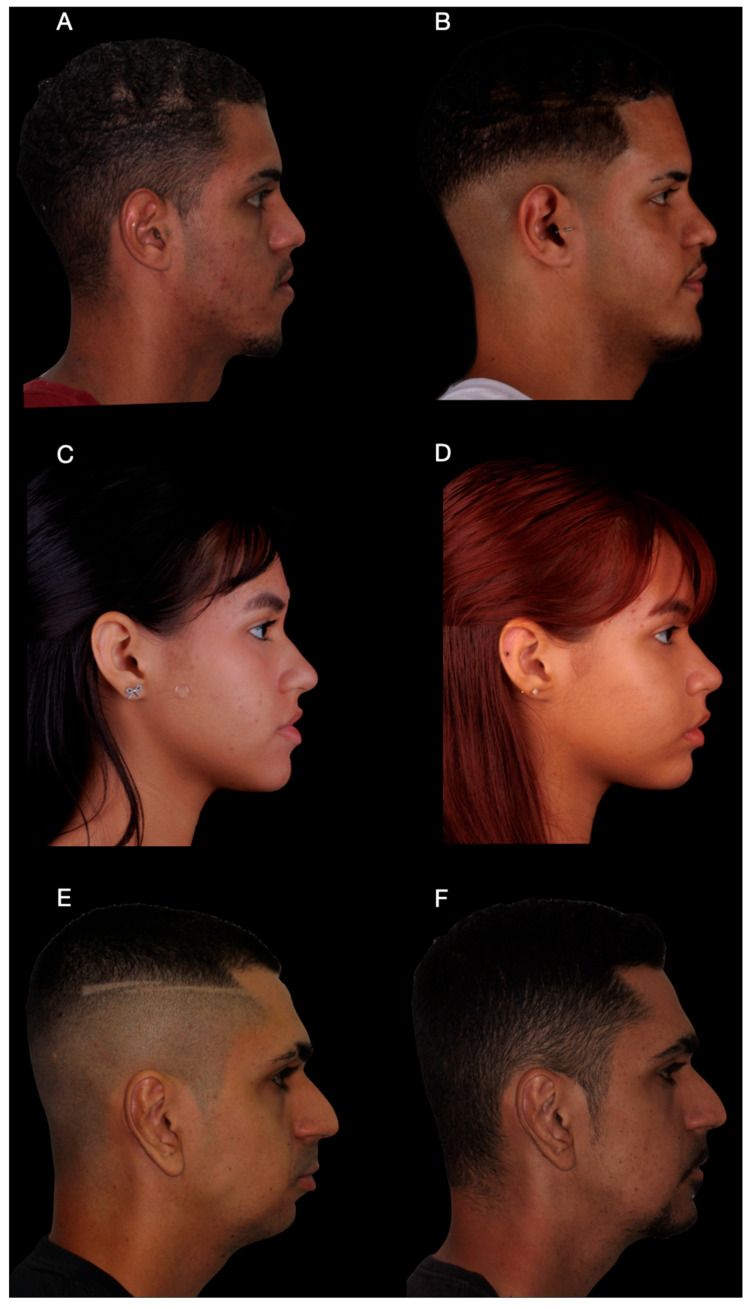
Profile clinical photographs before and after surgery. (**A**,**B**) Patient 1, profile view before surgery and at 12-month follow-up, demonstrating improved balance of the lower facial third. (**C**,**D**) Patient 2, profile view before surgery and at 12-month follow-up, showing appropriate chin positioning in relation to the lower lip. (**E**,**F**) Patient 3, profile view before surgery and at 12-month follow-up, illustrating correction of mandibular retrognathism and anterior repositioning of the chin.

Despite the asymmetry and combined surgical approach, the mentoplasty phase required approximately 50 min of operative time, reflecting the increased complexity of asymmetric repositioning. Across the three cases, operative time for the mentoplasty procedures ranged from 40 to 50 min, with a mean duration of approximately 45 min. All patients were followed for a minimum of 12 months postoperatively (Figure 7 and Figure 8). Clinical examination and postoperative CBCT were used to assess healing, complications, and the accuracy of chin positioning. No major complications were observed. Transient paresthesia occurred in all cases, being moderate in Case 1 and mild in Cases 2 and 3, with complete resolution within the first postoperative month.

Descriptive linear comparisons between planned and postoperative CBCT measurements are summarized in Table 1 and suggest consistency between the virtual plan and the achieved surgical outcome.

### 2.5. Fabrication of 3D-Printed Positioning and Cutting Guides

Three-dimensional surgical guides (positioning and cutting guides) were designed from anatomical models and exported as STL files. The models were processed using Anycubic Photon Workshop software version 3.6.2 (Shenzhen Anycubic Technology Co., Ltd., Shenzhen, China) to optimize orientation and support structure placement, minimizing printing deformation. Guide fabrication employed vat photopolymerization technology (Anycubic Photon M7 Pro 3D printer; Anycubic^®^, Shenzhen, China) with proprietary resin, using a 0.05 mm layer thickness and 2 s/layer exposure time per manufacturer’s guidelines to achieve high precision and optimal material curing [12].

Metal sleeves were not required due to the limited drilling depth and the use of low-speed drilling under continuous saline irrigation and suction. Additionally, the guides were printed using purple-colored resin, which facilitates visual identification of any potential debris if released intraoperatively. Furthermore, the cutting guide was intentionally designed with a short profile, serving only to guide the initial osteotomy trajectory rather than the full depth of the cut. This design reduces direct contact between the saw blade and the guide, minimizing the risk of guide damage and polymer debris release during osteotomy execution. Post-processing included a 5 min 99% isopropyl alcohol wash to remove residual resin, followed by a UV post-curing (405 nm, 10 min) to finalize polymerization and improve mechanical properties. All fabrication steps were conducted under controlled laboratory conditions (22–24 °C) to maintain consistency and encouraging agreement [12] (Figure 1).

## 3. Discussion

Customized surgical guides have demonstrated clinical relevance in the treatment of mandibular asymmetries by improving control over the three-dimensional positioning of the osteotomized segment when compared with conventional freehand techniques [13]. Given that chin contour and projection play a decisive role in facial harmony, patient-specific planning is particularly relevant even in isolated mentoplasty procedures [13]. Assis et al. reported the use of two distinct guides for osteotomy and fixation, enabling accurate transfer of virtual planning in cases of marked mandibular deviation [14]. Similarly, Olszewski et al. described the combined use of three-dimensional cephalometry, rapid prototyping, and customized guides as an effective strategy to enhance predictability and technical control in complex genioplasty procedures [15].

The use of personalized guides has been associated with operative advantages in selected clinical scenarios. Antúnez-Conde Hidalgo et al. reported a reduction in mean operative time in guided genioplasty compared with conventional techniques, with particularly noticeable effects in cases involving asymmetric mentonian movements [16]. In a larger series of 23 cases, Arcas et al. [17,18] reported high aesthetic predictability, reduced surgical complexity, shorter operative times, and low complication rates when customized guides and plates were used. While these reports suggest potential benefits related to workflow organization and intraoperative guidance, such findings should be interpreted within the context of heterogeneous study designs and variable outcome measures.

From a technical standpoint, the literature consistently emphasizes the role of guide-assisted approaches in improving control over bone positioning. Olszewski et al. demonstrated that prototype-based guides facilitate not only accurate osteotomies but also preoperative adaptation of fixation plates on physical models, contributing to intraoperative stability [15,19]. This level of control becomes particularly relevant in extensive or anatomically challenging osteotomies, such as mini-wing mentoplasty. As described by Cordier et al., such techniques may be associated with increased technical complexity due to anatomical constraints, reinforcing the value of precise intraoperative reference systems [20]. In this context, customized guides may contribute to safer and more controlled execution of planned movements.

Although concepts derived from minimally invasive orthognathic surgery have been discussed in the literature, the present case series does not aim to demonstrate or validate a minimally invasive surgical technique. Instead, the design concept applied to the surgical guides focused on optimizing guide geometry, segmentation, and support to facilitate placement under limited surgical access, independent of the specific mentoplasty technique employed. Design features such as occlusal-supported drilling orientation guides and compact bone-supported cutting guides illustrate how patient-specific solutions can improve workflow organization without implying reduced surgical exposure or morbidity [7,8,9,10,11].

The adoption of in-house fabrication for customized surgical guides has been proposed as a strategy to improve access to digital surgical planning, particularly within public healthcare systems. However, it is essential to recognize that in-house production shifts part of the resource burden from industry to the clinical team and institution. While direct material consumption may be low, overall implementation costs remain highly context-dependent and include planning time, personnel training, post-processing, quality control, and sterilization logistics [9,18].

Vat photopolymerization technology offers advantages in terms of surface resolution and geometric fidelity when compared with fused deposition modeling (FDM) and selective laser sintering (SLS) for the fabrication of patient-specific surgical guides [9,21,22]. Photopolymer resins indicated for surgical guide manufacturing have demonstrated adequate performance when appropriate post-processing and sterilization protocols are followed [23,24]. In addition, low-temperature hydrogen peroxide plasma sterilization has been shown to preserve dimensional stability of three-dimensionally printed guides, supporting their clinical applicability when material-specific protocols are respected [25].

In the present case series, vat photopolymerization enabled rapid in-house production of cutting and positioning guides with low direct material consumption and short fabrication times. Nevertheless, these findings should be interpreted as an analysis of direct material usage rather than a comprehensive economic evaluation.

Several limitations must be acknowledged. This study represents a small case series without a control group, limiting generalizability. Although postoperative imaging suggested correspondence between planned and achieved chin positioning, the absence of standardized accuracy metrics, three-dimensional superimposition analyses, and validated patient-reported outcome measures restricts the strength of any conclusions regarding accuracy or clinical benefit. Furthermore, the in-house workflow described represents an institution-specific approach and does not replace formal regulatory validation or a comprehensive quality management system. Future investigations should focus on larger cohorts, standardized outcome measures, and systematic evaluation of governance and quality assurance pathways.

The values presented refer exclusively to direct material consumption and printing time (Table 2). Indirect costs such as software licensing, equipment depreciation, personnel time for planning and design, post-processing, quality control, and sterilization logistics were not included and may significantly influence total implementation costs.

## 4. Conclusions

Within the limitations of a small case series, this study demonstrates the technical feasibility of using customized, in-house-fabricated cutting and positioning guides for the correction of chin asymmetry. The described workflow enabled consistent transfer of virtual planning to the operative field and facilitated intraoperative orientation during mentoplasty procedures.

Low direct material consumption and short fabrication times highlight the potential accessibility of vat photopolymerization for institutional implementation. However, these findings should be interpreted as exploratory and descriptive rather than as evidence of clinical superiority, reduced invasiveness, or validated accuracy. Further studies involving larger samples, standardized quantitative assessments, and structured governance frameworks are required to better define the role of in-house manufactured surgical guides in mentoplasty.

## Figures and Tables

**Table 1:** Planned versus achieved linear chin movements (mm) at predefined reference points.

Case	Reference Point	Planned (mm)	Postoperative CBCT (mm)	Absolute Deviation (mm)
	Right canine	5.50	5.24	0.26
**1**	Midline	7.00	7.05	0.05
	Left canine	5.20	5.28	0.08
	Right canine	2.60	2.00	0.60
**2**	Midline	2.00	2.20	0.20
	Left canine	2.40	1.70	0.70
	Right canine	5.50	5.43	0.07
**3**	Midline	6.30	6.39	0.09
	Left canine	6.80	6.82	0.02

**Table 2:** Printing parameters, resin consumption, and fabrication time for customized surgical guides in each case.

Patient	Resin Volume (mL)	Print Time (min)	Material Cost (US$)
First case	8.0	40	2.50
Second case	5.2	41	1.80
Third case	4.2	43	1.50

## Data Availability

The data presented in this study are available on request from the corresponding author due to privacy concerns.

## Abbreviations

The following abbreviations are used in this manuscript:

3D	Three-dimensional
CASS	Computer-Aided Surgical Simulation
STL	Standard Tessellation Language
DICOM	Digital Imaging and Communications in Medicine
BMM	Bone-Mounted Method
CBCT	Cone Beam Computed Tomography
TMJ	Temporomandibular Joint
FDM	Fused Deposition Modeling
SLS	Selective Laser Sintering
PA12	Polyamide 12
UV	Ultraviolet
CAAE	Certificate of Approval for Ethical Assessment
UNIFESO	Centro Universitário Serra dos Órgãos
